# Reaction of the Liver upon Long-Term Treatment of Fluoxetine and Atorvastatin Compared with Alcohol in a Mouse Model

**DOI:** 10.1155/2021/9974969

**Published:** 2021-12-30

**Authors:** Zhiliang Chen, Tony C. H. Chow, Shicong Wang, Gigi C. T. Leung, Sharon L. Y. Wu, David T. Yew

**Affiliations:** ^1^Fujian Provincial Key Laboratory of Pien Tze Huang Natural Medicine Research and Development, Zhangzhou Pien Tze Huang Pharmaceutical Co., Ltd, Fujian 363000, China; ^2^School of Chinese Medicine, The Chinese University of Hong Kong, Hong Kong, China; ^3^Hong Kong College of Technology, Hong Kong, China

## Abstract

**Background:**

Alcoholism is known to cause liver toxicity and is extensively researched. On the other hand, stress, depression, and obesity are interrelated conditions with alcoholism, and their medications would affect the liver itself. In this study, we investigated the effects of the drugs fluoxetine and atorvastatin on the liver and compared with those of alcohol in a mouse model.

**Methods:**

Comparisons of animals treated with the three drugs were carried out: serum aspartate transaminase (AST), alanine transaminase (ALT), and albumin were measured; liver tumor necrosis factor alpha (TNF alpha) and transforming growth factor beta (TGF beta-1) levels were evaluated; proliferative cells were detected via immunohistochemistry (IHC) targeting on proliferating cell nuclear antigen (PCNA) and minichromosome maintenance complex component 2 (MCM2); for apoptosis, IHC targeting on activated caspase-3 and terminal deoxynucleotidyl transferase dUTP nick end labeling (TUNEL) were employed; and histopathology was also documented in all groups.

**Results:**

For ALT, AST, albumin, and liver TNF alpha, only the ethanol group surged to significantly higher levels. For TGF beta-1, both ethanol and atorvastatin groups reached a significantly higher level. PCNA and MCM2 showed increased proliferation in the livers of all three groups, with the ethanol group having the highest number of positive cells followed by atorvastatin and then the fluoxetine group. As for cell death, both ethanol and fluoxetine groups showed significantly more apoptosis than control in TUNEL and activated caspase-3, while in the atorvastatin group, activated caspase-3 positive cells increased significantly, but the increase in TUNEL-positive cells did not reach statistical significance.

## 1. Introduction 

Alcoholism, especially chronic alcoholism, has been a long-existing health problem, with hepatic injuries being especially common as the liver is the primary site of alcohol metabolism. For many years, research studies have clarified alcoholism into several stages including inflammatory insults within various immune cells, fatty degeneration, cell death, and finally fibrosis [1]. The oxidative stage was, perhaps, the primary step [2], while the products of acetaldehyde played an undeniable role [3, 4], along with other free radicals [5]. Fatty degeneration became a hallmark of early injury [6]. Downregulation of glutathione reductase and cytochrome P415 was an accompanying feature [7]. Cell death followed, while invading immune cells and stellate cells, as well as Kupffer cells, helped in the formation of fibers leading to cirrhosis [1]. Such liver damages cause a rise of blood transaminase levels such as alanine transaminase and aspartate transaminase (ALT and AST), and they serve as important markers for liver damage [8]. Proliferation and apoptosis initiated in the liver have been documented in the literature after toxicity (alcohol and CCL4) [9, 10] and after inflammation [11] or other organic insults (e.g., bile ligation) [11, 12]. Toxicity of the liver would lead to cell death of both necrosis and apoptosis. While necrosis was well understood, apoptosis in relation to alcohol and therapeutic drugs was less defined and needs clarification [13]. In addition, alcohol intoxication also leads to other well-documented changes in fibrosis and inflammation [14].

With these well-studied pathways, alcoholism has become a golden standard of chronic liver damage. On the other hand, with the burden of the present world, stress and obesity are stigmas of society, and they are interrelated conditions with alcoholism [15]. Individuals suffering from these conditions seek help on antidepressants such as fluoxetine, while those prone to high cholesterol use drugs such as atorvastatin. As a result, a lot of patients are now taking atorvastatin and fluoxetine daily. Although pronounced as safe by the drug companies, these drugs themselves would still affect the liver itself, causing inflammation, degeneration, fibrosis, proliferation, and apoptosis of liver cells [16, 17]. The toxicity of the latter needs an in-depth investigation, especially with animal models. In the present work, we used a mouse of chronic alcohol intoxication with weekly acute boosting of binge alcohol [18], to serve as a gold standard or baseline of the toxic liver, and compared this model with mouse models of antidepressant intoxication and anticholesterol intoxication to delineate the level of toxic effects. The present evaluation included ALT, AST, albumin, TNF alpha, TGF beta-1, proliferation (PCNA and MCM2), and apoptosis (TUNEL and caspase-3 targeting on two different stages of apoptosis). Histopathology on fibrosis and fatty degeneration was also compared.

## 2. Materials and Methods

### 2.1. Experimental Animals

Animal experiments of this study were approved by the Research Ethics Review Panel for Animal Experiments of Hong Kong College of Technology. Twelve-week-old ICR mice (*Mus musculus*) used in the study were provided by the Laboratory Animal Services Centre of the Chinese University of Hong Kong. All animals were kept in a room maintained at 22 ± 2°C and in 12 : 12 hour light-dark cycles. Twenty mice were randomly divided into four groups, ethanol (*n* = 5), fluoxetine (*n* = 5), atorvastatin (*n* = 5), and control groups (*n* = 5). Water and corresponding diets were available *ad libitum*.

The ethanol group was fed on a regular diet mixed with ethanol. The percentage of ethanol was 5% (w/v). On average, 21.25 g ethanol/kg/day ethanol was consumed by mice through a liquid diet. Mice were gavaged (IG) with ethanol (5 g/kg body weight) every five days in the 60-day feeding period.

Fluoxetine, atorvastatin, and control groups were fed on a regular diet. Fluoxetine, atorvastatin, and control groups were gavaged with fluoxetine (24.6 mg/kg body weight), atorvastatin (24.6 mg/kg body weight), and normal saline every day for 60 days, respectively.

After the 60-day treatment, mice were anesthetized by inhalation of 1% isoflurane and blood samples were collected by cardiac puncture. Mice were then euthanized by cervical dislocation. Blood samples were allowed to clot at room temperature for 30 minutes. The clot was removed by centrifugation of the samples at 2000 g for 10 minutes at 4°C. Serum samples were stored at −20°C. The liver was excised and fixed in 10% phosphate-buffered formalin. To avoid regional differences, all samples were taken from the middle portion of the right lobe.

### 2.2. Serum Aspartate Transaminase (AST) and Alanine Transaminase (ALT) Activity Assay

AST and ALT activity assay were performed using the Aspartate Aminotransferase (AST or SGOT) Activity Colorimetric Assay Kit (BioVision, Milpitas, USA) and Alanine Aminotransferase (ALT or SGPT) Activity Colorimetric/Fluorometric Assay Kit (BioVision, Milpitas, USA), respectively. Procedures and calculations followed the manual provided by the manufacturer. The provided reaction mix was added to the serum samples. Initial OD (450 nm for AST and 570 nm for ALT) and OD after incubation at 37°C for 1 hour were measured. With the absorbance of the generated reaction product and standard curve, the enzyme activities were calculated.

### 2.3. Serum Albumin Assay

Albumin assay was performed using the BCG Albumin Assay Kit (Merck, Darmstadt, Germany). Procedures and calculations followed the manual provided by the manufacturer. After incubating samples with the reaction reagent, OD at 620 nm was measured and the amount of albumin was calculated.

### 2.4. Preparation of Histological Samples

Fixed liver samples were dehydrated in graded alcohol, cleared in xylene, and embedded in paraffin. Paraffin block was sectioned into 6 *µ*m-thick sections and mounted on slides. The sections were deparaffinized in xylene and rehydrated in graded alcohol before staining.

### 2.5. Hematoxylin and Eosin (H&E) Stain and Sirius Red Stain

For H&E stain, the rehydrated sections were stained in Mayer's hematoxylin solution for 5 minutes, differentiated in acid alcohol, blued in running tap water, and stained in eosin solution for 2 minutes. The sections were then dehydrated in alcohol, cleared in xylene, and mounted in dibutylphthalate polystyrene xylene (DPX). A histopathological study was made under a bright-field microscope.

Sirius red stain was used to detect collagen in the liver tissue. The rehydrated sections were stained with Picrosirius red solution for 60 minutes. After rinsing in two changes of acetic acid solution and absolute alcohol, the sections were dehydrated in two changes of absolute alcohol, cleared in xylene, and mounted in DPX.

### 2.6. Immunohistochemistry (IHC)

After heat-induced antigen retrieval in boiling sodium citrate buffer for 20 minutes, the rehydrated sections were incubated in phosphate buffer saline (PBS) with 10% goat normal serum and 1% bovine serum albumin for blocking. The sections were then incubated in anti-PCNA (Merck, Darmstadt, Germany), anti-MCM2 (Abcam, Cambridge, UK), or anticleaved caspase-3 (Cell Signaling, Danvers, USA) rabbit polyclonal antibody overnight at 4°C. On the second day, after incubation in biotinylated goat anti-rabbit antibody (Abcam, Cambridge, UK) for 1 hour and streptavidin-horseradish peroxidase for 1 hour, 3, 3′-diaminobenzidine (DAB) substrate was added to sections. The reaction was stopped once the appropriate color was developed and the sections were counterstained in hematoxylin for 30 seconds, dehydrated, cleared in xylene, and mounted in DPX. The numbers of positive cells were quantified by counting positive cells in a 700 *μ*m2 field (100X). Three mice in each group and 4 fields from each mouse were acquired.

### 2.7. Terminal Deoxynucleotidyl Transferase dUTP Nick End Labeling (TUNEL)

TUNEL was performed using the ApopTag® Peroxidase In Situ Apoptosis Detection Kit (EMD Millipore, Temecula, USA) according to the manufacturer's instruction manual. Rehydrated sections were pretreated with proteinase K and quenched in 3% hydrogen peroxide. After incubated in terminal deoxynucleotidyl transferase (TdT) enzyme and digoxigenin-conjugated nucleotide for an hour, stop buffer was added to the sections to terminate TdT enzyme reaction. Sections were then incubated in the antidigoxigenin conjugate, incubated in 3, 3′-diaminobenzidine (DAB) for color development, dehydrated, cleared in xylene, and mounted in DPX. TUNEL was quantified by counting positive cells in a 700 *μ*m^2^ field (100X). Three mice in each group and 4 fields from each mouse were acquired.

### 2.8. Enzyme-Linked Immunosorbent Assay (ELISA)

The snap-frozen liver samples were homogenized in ice-cold phosphate-buffered saline (PBS) with protease and phosphatase inhibitors. The homogenate was centrifuged at 15000 rpm at 4°C for 10 minutes, and the supernatant was retained. Tumor necrosis factor alpha (TNF alpha) and transforming growth factor beta 1 (TGF beta-1) in liver homogenate were quantified by commercially available TNF alpha (500850, Cayman Chemical, Ann Arbor, USA) and TGF beta-1 (ab119557, Abcam, Cambridge, UK) ELISA kits under the instructions of the manufacturers.

### 2.9. Statistical Analysis

Analysis was performed using the GraphPad Prism7.0 software (GraphPad Software Inc., USA). The results are presented as means ± SD. The significance of intergroup differences was estimated by one-way analysis of the variance (ANOVA), followed by a post hoc Tukey test.

## 3. Results

**Figure 1:**
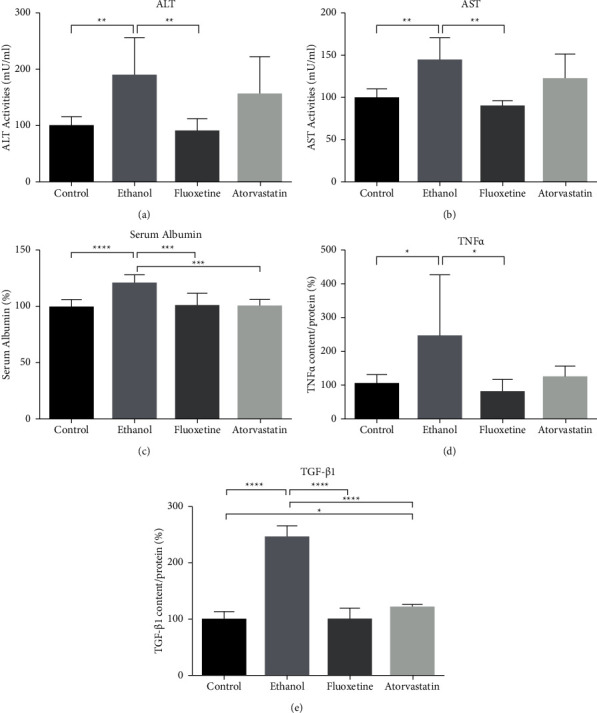
ALT activity (a), AST activity (b), serum albumin level (c), TNF alpha level (d), and TGF beta 1 level (e) of saline-, ethanol fluoxetine-, and atorvastatin-treated mice, respectively. Results are shown as mean ± SD; ^*∗*^ denotes *P* ≤ 0.05; ^*∗∗*^ denotes *P* ≤ 0.01; ^*∗∗∗*^ denotes *P* ≤ 0.001; and ^*∗∗∗∗*^ denotes *P* ≤ 0.0001.

ALT and AST assays revealed that, in the ethanol group, serum ALT and AST levels were higher than in control, while the higher activity levels of the atorvastatin group were not significantly different from the activity levels of the control group (Figures 1(a) and 1(b)). The fluoxetine group had serum ALT and AST levels similar to the control group (Figures 1(a) and 1(b)). Results of serum albumin, TNF alpha, and TGF beta-1 assays are presented in Figures 1(c), 1(d), and 1(e), respectively. Serum albumin level was slightly higher in the ethanol group (Figure 1(c)). TNF alpha and TGF beta-1 levels of ethanol and atorvastatin groups were higher than in the control group, while the increase of TNF alpha level in the atorvastatin group did not reach statistical significance (Figures 1(d) and 1(e)).

**Figure 2:**
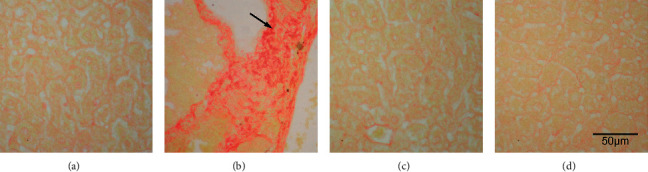
Sirius red stain of the saline- (a), ethanol- (b), fluoxetine- (c), and atorvastatin-treated (d) liver. Fibrosis (arrow) was noted in the ethanol-treated liver (b), 400X.

**Figure 3:**
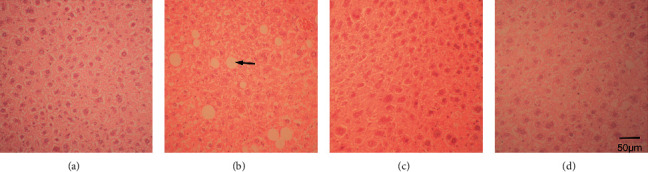
H&E stain of the saline- (a), ethanol- (b), fluoxetine- (c), and atorvastatin-treated (d) liver. The arrow denotes one of the lipid vesicles in fatty degeneration in the ethanol-treated liver (b), 200X.

Histopathology showed significant fibrosis in the ethanol group (Figure 2(b)) versus the control (Figure 2(a)), fluoxetine (Figure 2(c)), and atorvastatin groups (Figure 2(d)) which had no significant fibrosis. Lipid vesicles in fatty degenerations were seen in the ethanol group (Figure 3(b)) while absent in the control, fluoxetine, and atorvastatin groups (Figures 3(a), 3(c), and 3(d)). Fatty degeneration of the liver is the primary degeneration of the liver signifying the production and accumulation of lipid vesicles due to factors such as lipid metabolism problems, alcoholism, and drug toxicity [19].

**Figure 4:**
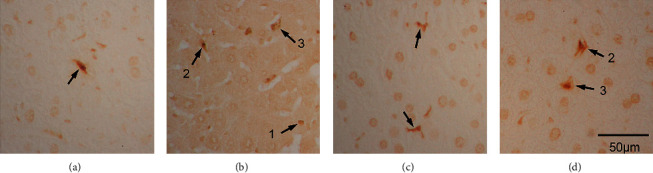
TUNEL-positive cells in the saline- (a), ethanol- (b), fluoxetine- (c), and atorvastatin-treated (d) liver. Arrows denote positive cells; 1 denotes a possible positive liver cell; 2 denotes a possible Kupffer cell; and 3 denotes a possible stellate cell, 400X.

**Figure 5:**
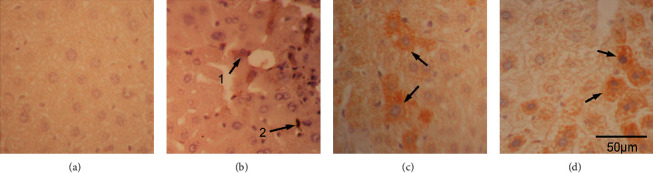
Activated caspase-3-positive cells in the saline- (a), ethanol- (b), fluoxetine- (c), and atorvastatin-treated (d) liver. Arrows denote positive cells; 1 denotes a positive liver cell; and 2 denotes a Kupffer cell, 400X.

Microscopic inspection revealed that, for apoptotic cell death, both TUNEL- and activated caspase-3-positive liver parenchymal cells and the Kupffer/stellate cells of the ethanol group (Figures 4(b) and 5(b)) were more numerous than in the control group (Figures 4(a) and 5(a)). Apoptotic cells were also prominent in the fluoxetine group (Figures 4(c) and 5(c)). In the atorvastatin group, both TUNEL-positive cells and activated caspase-3-positive cells were present (Figures 4(d) and 5(d)), while activated caspase-3-positive cells, in this case, were mainly parenchymal liver cells and TUNEL-positive cells were mainly Kupffer/stellate cells.

**Figure 6:**
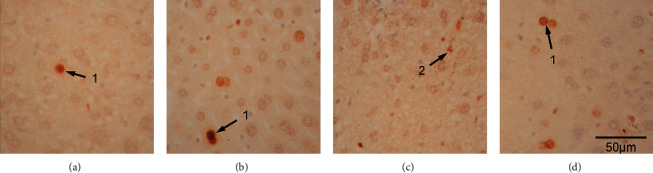
PCNA-positive nuclei in the saline- (a), ethanol- (b), fluoxetine- (c), and atorvastatin-treated (d) liver. Arrows denote positive cells; 1 denotes a positive liver cell nucleus; and 2 denotes a Kupffer cell nucleus, 400X.

**Figure 7:**
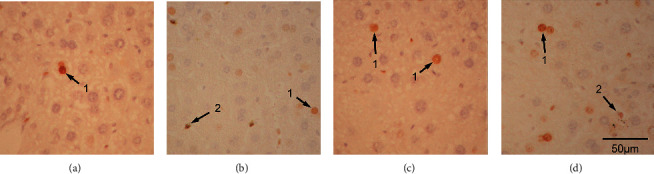
MCM2-positive nuclei in the saline- (a), ethanol- (b), fluoxetine- (c), and atorvastatin-treated (d) liver. Arrows denote positive cells; 1 denotes a positive liver cell nucleus; and 2 denotes a Kupffer or a stellate cell nucleus, 400X.

**Figure 8:**
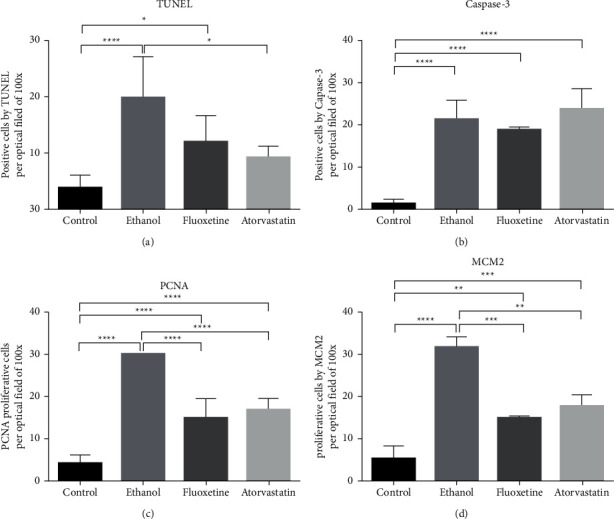
Positive-cells by TUNEL (a) and activated caspase-3 (b) and proliferative cells by PCNA (c) and MCM2 (d) per optical field of 100X. Results are shown as mean ± SD; ^*∗*^ denotes *P* ≤ 0.05; ^*∗∗*^ denotes *P* ≤ 0.01; ^*∗∗∗*^ denotes *P* ≤ 0.001; and ^*∗∗∗∗*^ denotes *P* ≤ 0.0001.

For proliferation, more PCNA- and MCM2-positive cells were observed in all treatment groups than in the control group (Figures 6(a) and 7(a)). Both PCNA- and MCM2-positive cells were found in both liver parenchymal cells and Kupffer/stellate cells of the ethanol group (Figures 6(b) and 7(b)). In the fluoxetine group, PCNA- or MCM2-positive cells were also substantial (Figures 6(c) and 7(c)) and most of them were Kupffer/stellate cells. In the atorvastatin group, there were a lot of PCNA- and MCM2-positive cells in both liver parenchymal and Kupffer/stellate cells (Figures 6(d) and 7(d)). Quantitative analysis of the density of apoptotic cells and proliferative cells per optical field of 100X in the four groups are depicted in Figure 8. Quantification was made with 3 animals in each group and 3 fields for each animal.

## 4. Discussion

Transaminase evaluation revealed a significant increase of both AST and ALT in the sera of the ethanol group and not in the fluoxetine and the atorvastatin groups. Serum albumin level is an indicator of liver function, and it was not much different in those groups. Only the ethanol group had a slightly higher albumin level than other groups, and it was probably not clinically relevant. TNF alpha is an important marker of inflammation, while TGF beta-1 is a marker of fibrosis. There were increases of TNF alpha in ethanol and atorvastatin groups, but only the ethanol group reached statistical significance. In contrast, there were significant increases of TGF beta-1 in both ethanol and atorvastatin groups.

An increase of proliferation in the liver reflected possible repair of tissue. Proliferation markers used in studies were proliferative cell nuclear antigen (PCNA) and minichromosome maintenance complex component 2 (MCM2). Each of these actually had different functions. PCNA was involved in forming a scaffold attracting proteins necessary for proliferation, for example, on DNA polymerase during proliferation and repair [20, 21]. On the other hand, MCM2 was associated with the unwinding of the double-strain DNA and proceeding with replication of the detached single strains [22]. MCM2 was initially detected from lymphoma cells and, thus, has a tumor origin [23]. PCNA was of plant origin [21]. The results of the two markers were compared as they could detect proliferative liver cells and Kupffer/stellate cells through two mechanisms. In our work, though with different mechanisms, we found that the trends in the results of the two markers were similar. This work recorded a prominent increase of proliferative cells in the ethanol group and the atorvastatin group, while in the fluoxetine group, MCM2 recorded less proliferating cells' increase in the liver. These reflected some forms of repair in liver tissues were possibly higher in ethanol and atorvastatin groups than in the fluoxetine group. The liver of the ethanol group was divided into 5 equal regions from bottom to top, and the relative percentage of PCNA proliferative cells was the highest in the bottom segment (roughly representing 30% of proliferative cells, unpublished data) and reflected the regional difference in proliferation. Our studies on the injured liver revealed that because of the difference of mechanism between the two markers and their action sites in proliferation, PCNA and MCM2 might have different absolute values depending on actual stages of proliferation.

For cell death, there are now new classifications of cell death in addition to apoptosis and necrosis, including necroptosis, cystoptosis (related to cytokines), lysosome cell death (related to cathepsin but could lead to necrosis or apoptosis), and many other forms [24]. Here, we limited ourselves to apoptosis (old programmed cell death). It is important to realize that even apoptosis did not relate only to injury and disease; it was also related to physiological stages such as hemostasis, remodeling, and aging.

In this study, TUNEL- and activated caspase-3-positive sites were found in both liver parenchymal and Kupffer/stellate cells. All groups had a significantly higher density of apoptotic cells than the control group, except for the atorvastatin group by TUNEL. It should be noted that TUNEL and activated caspase-3 again targeted different mechanisms of cell death and, thus, results would vary. Activated caspase-3 presence in the cells indicated the production of death enzyme for the cell (early stages of apoptosis), while TUNEL indicated the last stage of DNA fragmentation (late apoptosis), comparatively. Logically, detection of activated caspase-3 was of an earlier stage than the detection of DNA breaks during apoptosis. In our studies, the ethanol group had the highest number of TUNEL-positive cells. In the atorvastatin group, while TUNEL-positive cells were few, activated caspase-3-positive cells were of an all-time high. Cell death of early versus late stages in the liver in these groups would be one thing that clinicians had to watch if the results of mice studies could be extrapolated to humans.

Both proliferation and apoptosis in our experiments were the results of alcohol and drug injury. Proliferation is very much tied to regeneration and repair [25], while cell death included both apoptosis and necrosis. In this work, proliferation and cell death were both the highest in the ethanol group while those of the atorvastatin and fluoxetine groups were about the same. The eye-catching point was, however, in the trend of increase of transaminases, TNF alpha, and TGF beta-1 in the atorvastatin group over the fluoxetine group. It seemed atorvastatin was more toxic than fluoxetine and with toxicity second to alcohol. Other preliminary studies in our laboratory also showed necrosis of the liver in the three groups, but the amount was not excessive, with again alcohol toxicity leading the way (unpublished data).

## 5. Conclusions

On the whole, with the present dosages employed in this study and comparing alcohol, fluoxetine, and atorvastatin treatments, it was clear that ethanol caused the most damage, with changes in both ALT and AST. Fluoxetine had no rise in ALT and AST, while atorvastatin raised ALT and AST (insignificantly). ALT and AST were markers of liver damage. Overall, all groups treated with ethanol, fluoxetine, and atorvastatin resulted in increased proliferation and apoptosis. All experimental groups raised TUNEL-positive cells (late apoptosis) than the control group, while activated caspase-3-positive cells (early-stage apoptosis) were higher in the ethanol and atorvastatin groups. TGF beta-1 was raised in ethanol and atorvastatin groups signifying higher toxicity in both the groups in the animal models. Periodic monitoring of patients treated with these drugs is a must, even for long term.

## Data Availability

The data supporting the findings of this study are available within the article.

## Additional Points

*Research Highlights.* (1) PCNA and MCM2 showed increased proliferation in the livers of all three, ethanol, atorvastatin, and fluoxetine, groups. (2) Alcohol and fluoxetine groups showed significantly more apoptosis in the liver than control in the TUNEL and activated caspase-3 technique. (3) Alcohol and atorvastatin groups showed a significant increase in TGF beta-1 levels.
